# ASH2L‐K312‐Lac Stimulates Angiogenesis in Tumors to Expedite the Malignant Progression of Hepatocellular Carcinoma

**DOI:** 10.1002/advs.202509477

**Published:** 2025-07-29

**Authors:** Hexu Han, Shuai Wang, Lixing Ma, Haimeng Yin, Xinxiang Cheng, YiFan Wang, Suqin Xia, Yi Zhang, Yue Zhang, Rong Zhu, Cuixia Liu, Dakun Zhao, Xiangqian Gu, He Zhu, Yin Yuan

**Affiliations:** ^1^ Department of Gastroenterology The Affiliated Taizhou People's Hospital of Nanjing Medical University Taizhou School of Clinical Medicine Nanjing Medical University Taizhou Jiangsu 225300 People's Republic of China; ^2^ Clinical Medical Laboratory Center The Affiliated Taizhou People's Hospital of Nanjing Medical University Taizhou Jiangsu 225300 China; ^3^ Department of Hepatobiliary Surgery Changzhi People's Hospital The Affiliated Hospital of Changzhi Medical College No. 502 Changxing Middle Road Changzhi Shanxi 046000 China; ^4^ Department of Otorhinolaryngology Head and Neck surgery Affiliated Hospital of Nantong University Medical School of Nantong University Nantong 226001 China; ^5^ Department of General Surgery of the Wuxi NO.2 Chinese Medcine Hospital Wuxi Jiangsu 214000 China; ^6^ Department of Hepatobiliary Surgery The Affiliated Wuxi People's Hospital of Nanjing Medical University Wuxi People's Hospital Wuxi Medical Center Nanjing Medical University Wuxi 214000 China; ^7^ Drug Clinical Trial Center The Affiliated Taizhou People's Hospital of Nanjing Medical University Taizhou 225300 China; ^8^ Department of Hepatobiliary Surgery The Affiliated Taizhou People's Hospital of Nanjing Medical University Taizhou School of Clinical Medicine Nanjing Medical University Taizhou Jiangsu 225300 People's Republic of China

**Keywords:** angiogenesis, ASH2L, H3K4me3, HCC, lactylation, VEGFA

## Abstract

Hepatocellular carcinoma (HCC) is a common malignant tumor. However, the role of lactic acid–modified proteins in its pathogenesis is unclear. This study determines the distribution of a novel post‐translational modification—protein lactylation—in HCC to identify potential targets and obtain mechanistic insights into this disease. Using high‐throughput proteomics, lysine 312 lactylation (K312‐lac) of the Set1/Ash2 histone methyltransferase complex subunit (ASH2L) is revealed as a candidate for further investigation. Subsequently, alanyl‐tRNA synthetase 1 (AARS1) and histone deacetylase 1 (HDAC1) are shown to mediate lactylation modification of ASH2L. In vivo experiments demonstrate that ASH2L‐K312‐lac promotes HCC malignant progression and is positively correlated with tumor microvessel density, and vascular endothelial growth factor A (VEGFA) is identified as the key mediator in ASH2L‐K312‐lac‐induced angiogenesis. High‐throughput sequencing reveals ASH2L‐K312‐lac enrichment in the genome regions encoding *VEGFA*, facilitating targeted recruitment of the mixed lineage leukemia complex to these loci and enhancing *VEGFA* expression through synergistic activation of enhancers and promoters. Finally, clinical sample analyses and robust in vivo preclinical experiments identify ASH2L‐K312‐lac as a promising therapeutic target for clinical application. These findings provide a theoretical foundation for the clinical translation of ASH2L‐K312‐lac‐based treatment approaches, offering potential advancements in HCC diagnosis and treatment.

## Graphical abstract

In this study, ASH2L‐K312‐lac is identified as a critical driver that promotes the malignant progression of hepatocellular carcinoma (HCC). Furthermore, through a comprehensive series of cell biological and molecular biological experiments, integrated with diverse animal models and systematic application of high‐throughput sequencing technologies, it is demonstrated that ASH2L, an important epigenetic regulatory factor, undergoes lactylation modification at lysine residue 312. This post‐translational modification enhances VEGFA expression and secretion in HCC by synergistically activating promoter and enhancer elements, thereby facilitating hematogenous metastasis.

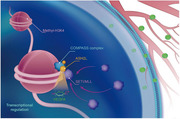

## 1 Introduction

Hepatocellular carcinoma (HCC) is one of the most prevalent malignant tumors, particularly in Asia and Africa.^[^ ^1^ ^]^ According to the World Health Organization, approximately 841 000 new cases of HCC are reported annually worldwide.^[^ ^2^ ^]^ The National Cancer Center of China has identified HCC as the fourth most common cancer type and the third leading cause of cancer‐related deaths in China, with approximately 400 000 new cases and 300 000 deaths annually.^[^ ^3^ ^]^

Primary treatment modalities for HCC include surgical resection, liver transplantation, radiofrequency ablation, microwave ablation, interventional therapy (e.g., transarterial chemoembolization), targeted drug therapy, and immunotherapy.^[^ ^4^ ^]^ Early HCC detection leads to better outcomes, improving the prospects for surgical resection or liver transplantation and markedly improving survival rates.^[^ ^5^ ^]^ However, there is a high prevalence of late‐stage diagnoses, and surgical options are limited in such cases,^[^ ^6^ ^]^ necessitating frequent reliance on interventional or drug therapy.^[^ ^7^ ^]^ Targeted drugs, such as sorafenib and lenvatinib, and immune checkpoint inhibitors,^[^ ^8^ ^]^ such as programmed cell death protein 1 and programmed death‐ligand 1, offer novel treatment options for patients who do not qualify for surgery.^[^ ^9^ ^]^ However, the efficacy of these medications varies among individuals and may lead to adverse effects. Therefore, studies have increasingly focused on identifying novel treatment avenues, including drugs derived from molecules naturally occurring in or produced by human cells.

Lactate is a by‐product of glycolysis and is primarily synthesized in muscle cells.^[^ ^10^ ^]^ Lactic acid is involved in energy metabolism and influences the acid–base balance, signal transduction, and the tumor microenvironment.^[^ ^11^ ^]^ Recent studies have improved the understanding of the multifaceted role of lactic acid,^[^ ^12^ ^]^ and it has been implicated in immune responses, neurophysiology, and cancer development.^[^ ^13^ ^]^ The development of lactate detection technology and the examination of the regulatory mechanisms underlying lactate metabolism have attracted substantial scientific interest.^[^ ^14^ ^]^ Lactate is a substrate for protein lactylation, which involves the covalent attachment of lactate to specific amino acid residues as a post‐translational modification mechanism. This modification alters protein structure and function,^[^ ^15^ ^]^ affecting stability, activity, subcellular localization, and molecular interaction.^[^ ^16^ ^]^ However, compared with well‐established post‐translational modifications, such as phosphorylation and acetylation, our understanding of the effects of protein lactylation remains limited. Although the roles of lactic acid and chemically modified proteins in HCC development and progression remain unclear, protein modification via lactylation may serve as a key therapeutic avenue for HCC treatment.

The Set1/Ash2 histone methyltransferase complex subunit (ASH2L) is a protein belonging to the chromatin modification complex that plays a crucial role in regulating gene expression,^[^ ^17^ ^]^ including DNA methylation and histone modification.^[^ ^18^ ^]^ ASH2L is a constituent of the Su(var)39, enhancer of zeste, trithorax (SET1)/mixed lineage leukemia (MLL) family—also known as the complex of proteins associated with Set1 (COMPASS) complex—which comprises a group of histone‐modifying enzyme complexes widely distributed in eukaryotes.^[^ ^19^ ^]^ Its primary function is to catalyze the methylation of histone H3 lysine 4 (H3K4), a modification crucial for various biological processes, including gene expression regulation, DNA replication, DNA repair, and chromatin remodeling.^[^ ^20^ ^]^ ASH2L is also involved in embryonic development, cell proliferation, and differentiation, and it has been implicated in the pathogenesis of certain diseases.^[^ ^21^ ^]^

We hypothesized that ASH2L lactylation enhances its binding ability to the genomic region encoding vascular endothelial growth factor A (*VEGFA*) and then recruits the MLL1 methyltransferase complex, thereby epigenetically regulating *VEGFA* expression. Thus, in this study, we aimed to analyze differentially modified proteins in HCC and adjacent tissues using mass spectrometry–based detection of protein lactylation modifications. The objective was to examine the levels of ASH2L lactylation in HCC specimens, exploring its involvement in HCC development and progression.

## 2 Results

### 2.1 Lysine‐312 Mediated ASH2L Lactylation

**Figure 1:**
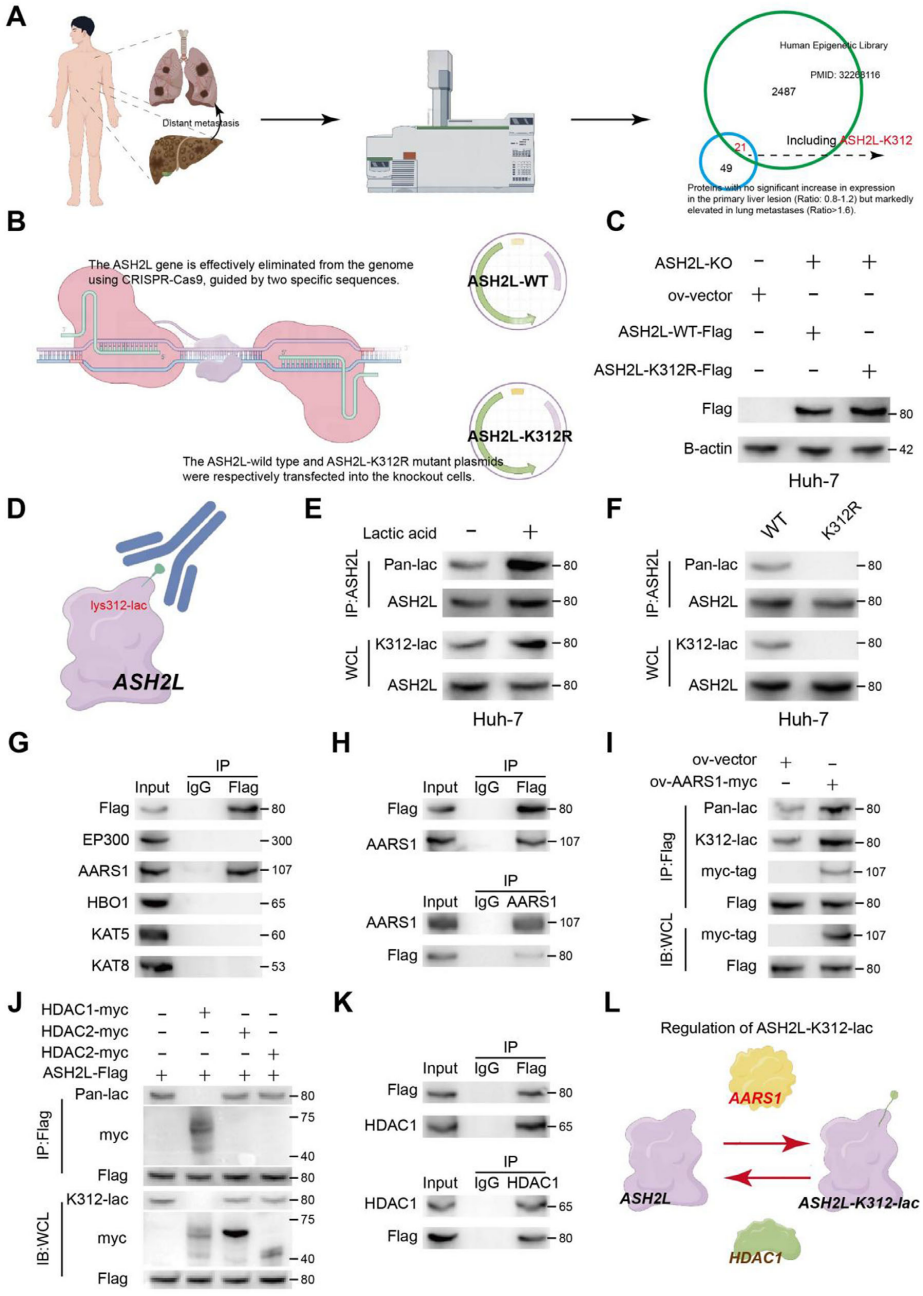
Lysine‐312 is the key residue that mediates ASH2L lactylation. a) High‐throughput lactylation‐modified omics analysis identified lysine‐312 as a potential lactylation site on the ASH2L protein. b) Development of ASH2L‐WT and ASH2L‐K312R cellular models. First, we used CRISPR‐Cas9 technology to eliminate ASH2L from genomic DNA. Subsequently, we separately introduced wild‐type (WT) and mutant ASH2L plasmids to establish ASH2L‐WT and ASH2L‐K312R cell lines, respectively. c) Western blotting revealed successful establishment of ASH2L‐WT and ASH2L‐K312R cell lines. d) Custom antibody developed to target lysine‐312 lactylation of the ASH2L protein (provided by Huaan Biotechnology Co., Ltd., Hangzhou, China). e) Following the direct addition of lactic acid (a final concentration of 100 mm) to the culture medium for 12 h, western blotting, and immunoprecipitation (IP) assays revealed a significant increase in the total lactylation and corresponding lysine‐312 lactylation levels in ASH2L. f) Western blotting and IP assays revealed a significant reduction in total lactylation and lysine‐312 autosomal lactylation levels in ASH2L in ASH2L‐K312R cells compared with ASH2L‐WT cells. g) Co‐IP screening assay performed to identify potential writers responsible for ASH2L lactylation. Only AARS1 interacted with ASH2L. h) Co‐IP assay revealed physical interaction between AARS1 and ASH2L. i) Western blotting and co‐IP assays revealed significantly elevated levels of total and lysine‐312‐specific lactylation of ASH2L following AARS1 overexpression. j) Western blotting and co‐IP assays revealed that HDAC1 was a delactylase that catalyzes the elimination of lactylation modification from ASH2L. k) Co‐IP assay revealed the physical interaction between HDAC1 and ASH2L. l) Schematic illustrating the synergistic regulation of ASH2L lactylation in hepatocellular carcinoma cells by AARS1 and HDAC1.

Upon reanalyzing our previously published dataset,^[^ ^22^ ^]^ we noted that the lactylation profiles across paired clinical tissue samples from three cohorts, comprising primary liver cancer, adjacent nontumorous tissue, and corresponding lung metastatic tumor tissue, exhibited distinct patterns (Figure S1A, Supporting Information). Key oncogenic signaling pathways, such as transforming growth factor beta, WNT, mammalian target of rapamycin, Hedgehog, angiogenesis, and MYC targets, were significantly upregulated in the primary and metastatic lesions, suggesting the potential role of protein lactylation in promoting HCC progression and distant metastasis (Figure S1A, Supporting Information). Based on these findings, we further investigated specific lactylated proteins potentially involved in metastasis. We systematically examined the differences in lactylated protein modifications between cancerous tissues and adjacent noncancerous tissues as well as between metastatic lesions and primary tumors. Integration of the findings from these two comparisons revealed that the protein sites were not significantly altered (ratio: 0.8–1.2) in the first comparison but showed significantly increased alterations in the second comparison (ratio: >1.6), indicating high‐risk modification sites potentially associated with hematogenous metastasis of HCC. We identified numerous lactylated proteins, among which components of the COMPASS complex, including ASH2L K312, were predominant. Considering the critical role of the COMPASS complex in chromatin modification and transcriptional regulation, we focused our investigations on ASH2L (**Figure** **1**A).

To verify whether ASH2L undergoes lactylation modification, we treated the cell culture medium with various lactic acid concentrations and subsequently analyzed the changes in ASH2L lactylation levels. The level of ASH2L lactylation increased significantly with either a longer exposure duration or higher concentrations (Figure S1B, Supporting Information). Subsequently, we developed an ASH2L‐K312R model to investigate the cellular inactivation of ASH2L by lysine 312 lactylation (K312‐lac). ASH2L knockout was performed in HCC cell lines, followed by transfection with FLAG‐tagged wild‐type (WT) ASH2L and ASH2L‐K312R plasmids (Figure 1B). Subsequently, HCC cell lines expressing ASH2L‐WT and the ASH2L‐K312R mutant were established (Figure 1C and Figure S1C, Supporting Information). To facilitate further evaluation, a custom antibody targeting the ASH2L‐K312‐lac modification was developed (Figure 1D). Next, to verify its role as a substrate facilitating ASH2L lactylation, the cell lines were treated with lactate (Figure 1E and Figure S1D, Supporting Information). Using the ASH2L‐K312‐lac antibody, K312 was identified as the sole site for ASH2L protein lactylation, validating the precise construction of the mutant cell line (Figure 1F and Figure S1E, Supporting Information).

Studying protein lactylation often requires examining the key enzymes that regulate its modification, such as writers and erasers. These enzymes are crucial for lactylation, and understanding their role provides a clearer picture of ASH2L lactylation regulation and its functional consequences in the context of this study. Therefore, we investigated the mechanisms underlying the dynamic equilibrium of ASH2L lactylation regulated by writers and erasers by performing an immunoprecipitation screening assay to identify the interacting proteins. The results showed that ASH2L only interacted with alanyl‐tRNA synthetase 1 (AARS1) (Figure 1G). This finding was corroborated by coimmunoprecipitation (co‐IP) assays performed using Huh7‐ASH2L‐WT cells (Figure 1H). To investigate the interaction between AARS1 and ASH2L‐mediated lactylation, Huh7‐ASH2L‐WT cells were transfected with a plasmid overexpressing AARS1, resulting in increased total and lys312‐specific lactylation levels of ASH2L (Figure 1I). These findings suggested that AARS1 was pivotal in mediating ASH2L lactylation. Yingming Zhao and colleagues at the University of Chicago identified Class I histone deacetylases (HDACs; HDAC 1–3) as the most effective erasers for lysine lactylation modifications in vitro. Furthermore, HDAC1 and HDAC3 exhibit demethylating activity in cellular contexts.^[^ ^23^ ^]^ Based on these experimental findings, we further investigated the key enzymes responsible for mediating ASH2L demethylation. Immunoprecipitation and western blotting revealed that HDAC1 was the primary enzyme mediating ASH2L delactylation within the cellular environment (Figure 1J, K). Our findings demonstrate that AARS1 promotes ASH2L lactylation and that HDAC1 mediates its delactylation. These two processes function synergistically, maintaining the dynamic equilibrium of the lysine 312 residue of ASH2L in a lactylated–delactylated state (Figure 1L).

### 2.2 ASH2L Lactylation Facilitated the Malignant Progression of HCC by Promoting Vascular Endothelial Cell Proliferation

**Figure 2:**
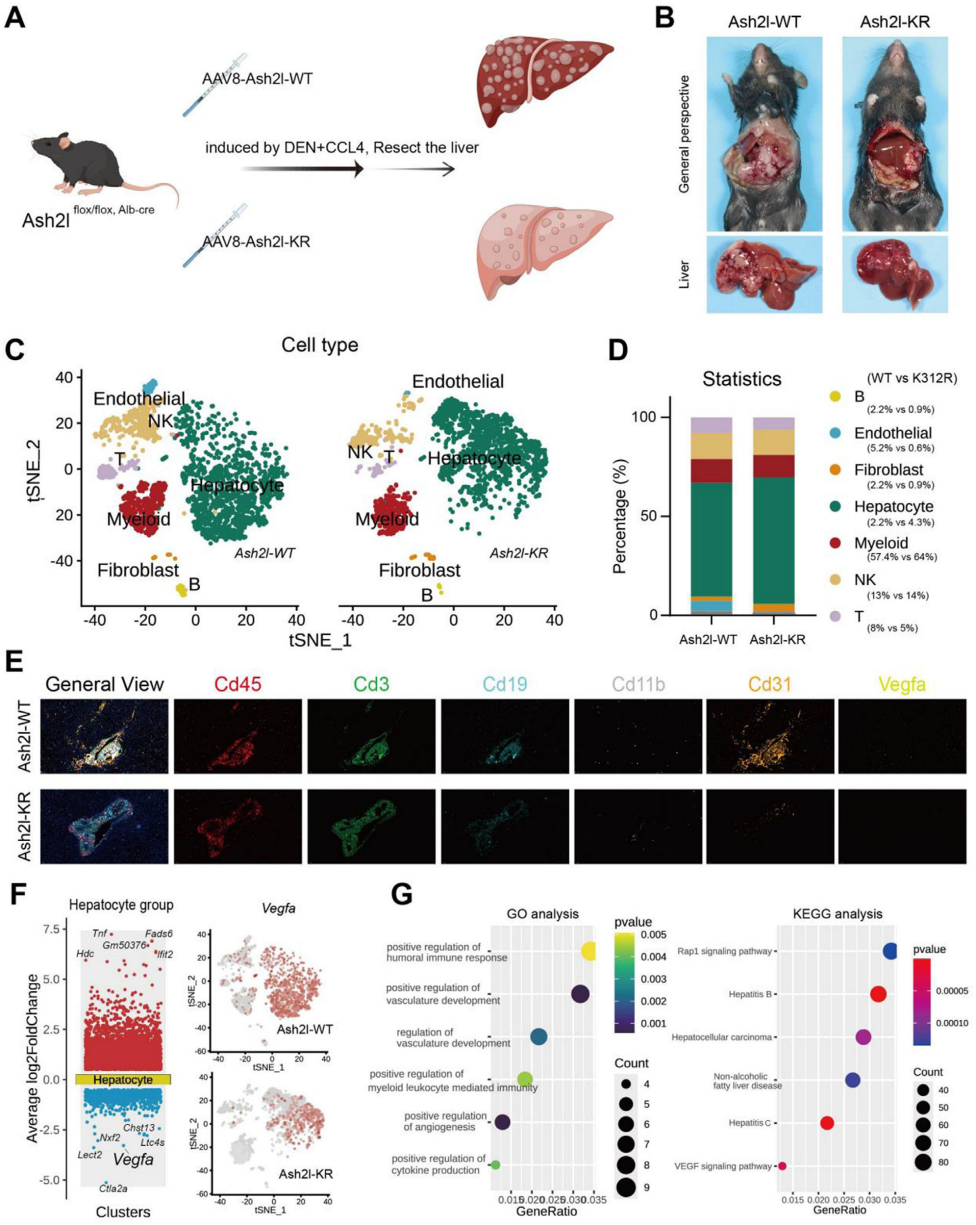
Lactylation of ASH2L facilitates the malignant progression of hepatocellular carcinoma (HCC) by promoting vascular endothelial cell proliferation. a) Adeno‐associated virus (AAV) tail vein injection in Ash2l^flox/flox, Alb‐cre^ mice and chemically induced (DEN+CCl_4_) HCC mouse models were used to analyze the effect of ASH2L‐K312‐lac on HCC progression (n = 5/group). b) Compared with Ash2l‐WT mice, mutant mice exhibited a significant reduction in tumor size and number following chemical induction of HCC (n = 5/group). c) Single‐cell RNA sequencing (scRNA‐seq) was performed on mouse liver cancer tissues obtained from the aforementioned experiments, followed by cell annotation and clustering. d) Cell annotation and clustering revealed a significant reduction in the proportion of endothelial cells in the mutant group compared with that in the Ash2l‐WT group. e) Multiplex immunohistochemical (mIHC) assays validated the observations derived from the scRNA‐seq‐based clustering analysis. f) Targeted analysis of the scRNA‐seq liver cell subpopulation data revealed that compared with the WT group, the mutant group contained a large number of genes with significant changes, including *Vegfa*. g) The differentially expressed genes (DEGs) identified above underwent GO enrichment and KEGG signaling pathway analysis, revealing their involvement in facilitating angiogenesis within HCC.

To further investigate the effect of ASH2L‐K312‐lactylation on HCC progression, we developed an animal model using genetically modified mice. In mice with liver‐specific deletion of *Ash2l*, liver‐specific expression of *Ash2l*‐WT or *Ash2l*‐K307R (the homologous site of lysine at position 312 of the corresponding human protein) was induced by adenovirus injection via the tail vein. We also established a mouse model with chemically induced HCC following the injection of N‐nitrosodiethylamine (DEN) and carbon tetrachloride (CCl_4_) (**Figure** **2**A). Following euthanization, we removed and weighed the livers of the mice, along with simultaneous AFP tests. There was a reduction in the number and size of liver tumors in the mutant group compared with those in the WT group (Figure 2B, n = 5 in each group). Furthermore, the size of liver tumors and the extent of HCC progression were significantly lower in mutant mice than in WT mice (Figure S2A, Supporting Information), indicating that lactylation‐modified ASH2L promoted the malignant progression of HCC. To investigate the effect of ASH2L‐K312‐lac on HCC progression, we performed single‐cell RNA sequencing (scRNA‐seq) analysis of WT and mutant tumor tissues (Figure 2C). Categorizing and annotating the cells revealed notable levels of variation in the proportions of multiple cell types in the liver cancer samples from mutant mice, except for HCC cells (Figure 2D). We observed a slight reduction in the proportion of immune cell populations, particularly T cells and natural killer cells. In contrast, the proportions of myeloid cells, tumor‐associated fibroblasts, and B cells remained unchanged between the mutant and WT groups. Notably, the proportion of endothelial cells was significantly lower in the mutant group than in the WT group (Figure 2D). The findings of high‐throughput detection were further validated (indicated by CD31) by multiplex immunohistochemical (mIHC) staining of the liver cancer samples of mice (Figure 2E). Next, we performed a targeted analysis of the data on scRNA‐seq liver cell subpopulation. A substantial number of genes in the HCC parenchymal cells were notably altered in the mutant group compared with those in the WT group. Subsequent gene ontology (GO) enrichment and Kyoto Encyclopedia of Genes and Genomes (KEGG) pathway analyses revealed that the downregulated genes resulting from mutations were extensively involved in various biological processes within HCC, influencing multiple signaling pathways (Figure 2F,G). These findings supported the cell clustering and mIHC staining results, and *Vegfa* was identified as a major differentially expressed gene (DEG). Furthermore, the GO and KEGG analyses suggested that ASH2L‐K312‐lac influenced angiogenesis in HCC via the VEGFA signaling pathway. Considering that HCC is a malignant tumor rich in microvessels and that the VEGFA signaling pathway plays a key role, we hypothesized that ASH2L‐lys312 lactylation enhances angiogenesis, thereby facilitating HCC malignant progression.

### 2.3 ASH2L Lactylation Influenced Angiogenesis in HCC Tumor Cells Via VEGFA

**Figure 3:**
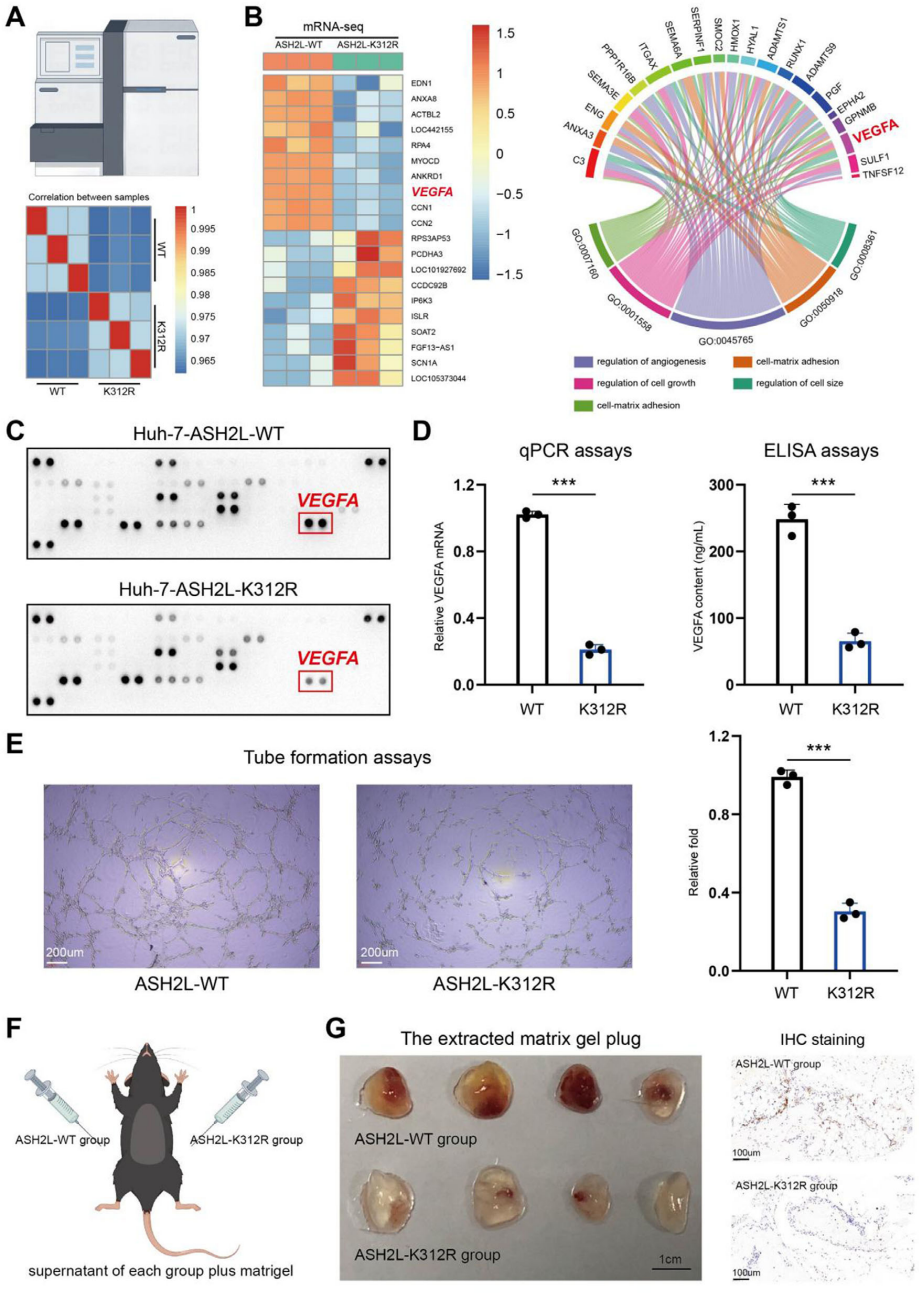
ASH2L lactylation affects angiogenesis in HCC tumor cells via VEGFA. a) The developed Huh7‐ASH2L‐WT and Huh7‐ASH2L‐K312R cells were subjected to transcriptome sequencing (bulk mRNA‐seq). b) Bulk mRNA‐seq data analysis showed that ASH2L‐K312‐lac effectively facilitated the upregulation of *VEGFA* expression to promote angiogenesis in HCC. c) Analysis using a Proteome Profiler Human Angiogenesis Antibody Array Detection Kit revealed reduced VEGFA secretion in Huh7‐ASH2L‐K312R cells. d) Quantitative polymerase chain reaction (qPCR) and enzyme‐linked immunosorbent assay (ELISA) results revealed reduced VEGFA expression and secretion in Huh‐7 cells with the K312R mutation. e) Tube formation assays revealed that the angiogenic potential of HUVECs to form capillary‐like structures in the supernatant of Huh7‐ASH2L‐K312R cells was significantly lower than that of Huh7‐ASH2L‐WT group. f) The supernatants of Huh7‐ASH2L‐WT and Huh7‐ASH2L‐K312R cells were separately combined with Matrigel for use in Matrigel plug assays in mice. g) Images of the retrieved Matrigel plugs. Compared with the Matrigel plug containing supernatant from the control group, those containing supernatant from the ASH2L‐K312R group showed more vascular infiltration (left), supporting the IHC results (right).

To further investigate the mechanism of ASH2L‐K312 lactylation, we performed transcriptome sequencing of Huh‐7‐ASH2L‐K312R cells and their corresponding control cells (**Figure** **3**A). Consistent with the scRNA‐seq findings, bulk mRNA‐seq revealed decreased *VEGFA* expression in the mutant Huh‐7 cells. Furthermore, GO functional enrichment analysis revealed that VEGFA was enriched with respect to the top five biological functions (Figure 3B). Because numerous cytokines facilitate HCC angiogenesis, we used a Proteome Profiler Human Angiogenesis Antibody Array Kit (ARY007, R&D Systems) to detect changes in the levels of different cytokines in the supernatants of WT and mutant cells. The results revealed that VEGFA was the only important angiogenesis‐regulating cytokine affected by ASH2L‐K312‐lac in HCC (Figure 3C). A more in‐depth analysis of the scRNA‐seq data identified fibroblasts and endothelial cells as the two most significant cell types interacting with hepatocytes (Figure S3A,B, Supporting Information). Because VEGFA is regulated by ASH2L lactylation in HCC cells (Figure 3A–C), we investigated the VEGFA signaling activity regulated by hepatocytes and its effects on endothelial cells (Figure S3C, Supporting Information). Notably, Ash2l‐KR significantly reduced the VEGFA signaling activity of hepatocytes and its effect on endothelial cells (Figure S3D, Supporting Information, P < 0.05). Furthermore, the interactions between Vegfa and its receptors (Vegfr1, Vegfr1r2, and Vegfr2) were significantly reduced in Ash2l‐KR compared with those in Ash2l‐WT (Figure S3E, Supporting Information). These findings support our conclusion that ASH2L lactylation promotes angiogenesis by enhancing VEGFA expression. To validate these findings, we performed quantitative polymerase chain reaction (qPCR) assays and enzyme‐linked immunosorbent assays (ELISAs) (Figure 3D and Figure S3F, Supporting Information). These results demonstrated that ASH2L‐K312‐lac was a critical lactylation modification site in HCC cells, facilitating VEGFA expression and secretion.

We performed in vivo and in vitro experiments to confirm the effect of ASH2L‐lys312 lactylation in HCC cells. Supernatants from Huh‐7‐ASH2L‐K312R and Huh‐7‐ASH2L‐WT cells in tube formation assays using human umbilical vein endothelial cells (HUVECs) were collected. The number of tube‐like structures formed by HUVECs in the conditioned medium derived from the mutant group was reduced compared with that of HUVECs in the conditioned medium derived from the Huh‐7‐ASH2L‐K312R group following the overexpression of *VEGFA* (Figure 3E and Figure S3G, Supporting Information). We also performed Matrigel plug assays in mice by separately combining the cell culture supernatants with the matrix gel (Figure 3F). The gel plugs were removed after 5 days, revealing a reduction in vascular infiltrates in the plugs mixed with the supernatant from the mutant group, and this was confirmed using immunohistochemical (IHC) staining (Figure 3G). Using previously established cell lines, we found that AARS1 regulated the expression levels of VEGFA in HCC cells. However, ASH2L‐lys312 exerted minimal effect on the cell cycle, apoptosis, and proliferation of tumor cells. Moreover, mutation at position 312 of ASH2L significantly diminished the ability of AARS1 to enhance VEGFA expression in HCC cells, indicating that AARS1 promoted angiogenesis in liver cancer through ASH2L lactylation (Figure S3H–K, Supporting Information). These findings demonstrated that ASH2L‐lys312 lactylation depends on VEGFA to promote angiogenesis in HCC.

### 2.4 ASH2L‐K312‐Lac Modification Caused an Increased Affinity for *VEGFA*, Facilitating its Transcription

**Figure 4:**
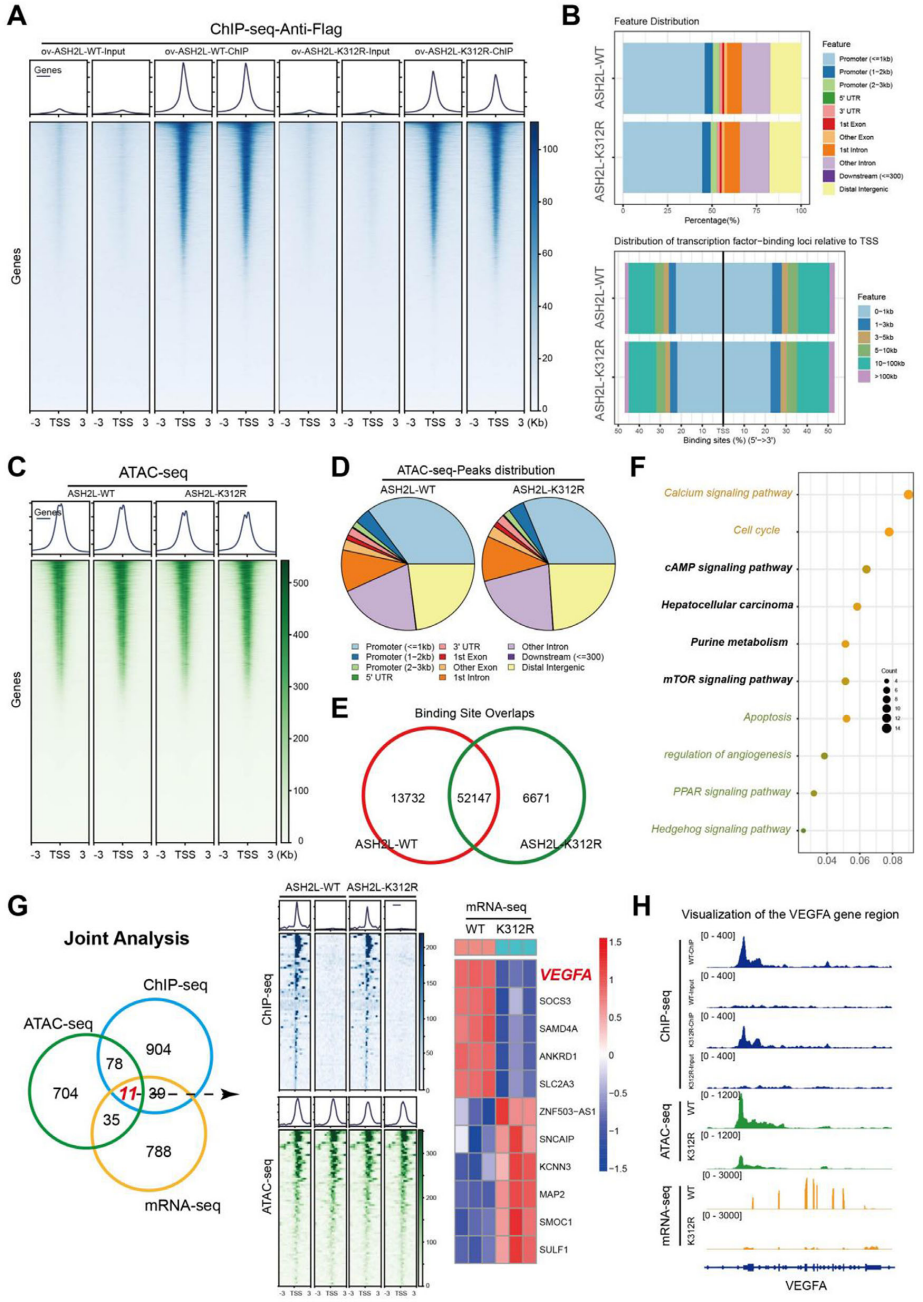
ASH2L‐K312‐lac exhibited increased affinity for *VEGFA*, facilitating its transcription. a) ChIP‐seq was used to investigate the genomic distribution of ASH2L in Huh7‐ASH2L‐WT and Huh7‐ASH2L‐K312R cells and to analyze its localization patterns. b) Analysis of the distribution disparities of ASH2L‐WT and ASH2L‐K312R across various chromatin elements revealed a significant reduction in the abundance of ASH2L‐K312R within the proximal promoter region. c) ATAC‐seq was used to examine alterations in chromatin accessibility in Huh7‐ASH2L‐WT and Huh7‐ASH2L‐K312R cells. d) The ATAC‐seq results indicated a significant reduction in open chromatin regions within the proximal promoter region of Huh7‐ASH2L‐K312R cells compared with Huh7‐ASH2L‐WT cells. e) Comparison of the disparities in open chromatin regions between Huh7‐ASH2L‐WT and Huh7‐ASH2L‐K312R cells. The overall openness decreased in the ASH2L‐K312R group, with approximately 10% of the peaks representing unique open regions. f) Comprehensive analysis of the unique open chromatin regions in Huh7‐ASH2L‐K312R cells indicated that they influenced various biological processes in HCC, including angiogenesis. g) Integrated analysis of ChIP‐seq, ATAC‐seq, and mRNA‐seq data revealed that ASH2L‐K312R regulates chromatin accessibility by directly binding to genomic DNA, thereby influencing the transcriptional regulation of specific genes, including *VEGFA*. h) IGV visualization validated the direct binding of ASH2L‐K312‐lac to the transcription start site (TSS) of the *VEGFA*‐encoding region, facilitating accessibility to this region and influencing *VEGFA* mRNA expression in HCC.

To further investigate how lactylation enhanced ASH2L‐mediated angiogenesis in HCC, we performed chromatin immunoprecipitation sequencing (ChIP‐seq) analysis of FLAG‐tagged WT and mutant ASH2L proteins in Huh‐7‐ASH2L‐K312R and Huh‐7‐WT cells to explore alterations in ASH2L binding sites across the genome (**Figure** **4**A). ASH2L‐K312‐lac showed preferential binding to the transcription start site located proximally to the upstream region of the gene promoter (Figure 4B).

Because promoter regions often exhibit changes in chromatin accessibility, we investigated this phenomenon in Huh‐7‐ASH2L‐K312R and Huh‐7‐ASH2L‐WT cells using assay for transposase‐accessible chromatin with sequencing (ATAC‐seq) (Figure 4C). Chromatin accessibility in Huh‐7‐ASH2L‐K312R cells, particularly within 1 kb of the transcription start site, was reduced in the promoter region (ASH2L‐WT vs. ASH2L‐K312R, 35.16% vs. 31.26%; chi‐square test, P < 0.001) (Figure 4D). Further examination revealed numerous coregulated binding sites shared between the WT and mutant groups within the open chromatin regions. In Huh‐7‐ASH2L‐K312R cells, unique open chromatin regions accounted for approximately 10% of its binding sites (Figure 4E). Functional annotation analysis revealed that the unique open chromatin regions regulated multiple biological processes in HCC, including angiogenesis (Figure 4F).

Considering the frequent association between alterations in chromatin accessibility and changes in gene expression, we performed an integrated analysis of ChIP‐seq, ATAC‐seq, and mRNA‐seq data, revealing that ASH2L‐lys312 lactylation directly affected 11 genes, including *VEGFA* (Figure 4G). Visualization using Integrative Genomics Viewer (IGV) showed reduced binding of ASH2L to the *VEGFA‐*encoding region in the genome of Huh‐7‐ASH2L‐K312R cells compared with WT cells. This was accompanied by a reduction in open chromatin regions and substantial downregulation of *VEGFA* expression (Figure 4H and Figure S4A, Supporting Information). Thus, ASH2L‐lys312 lactylation directly augmented *VEGFA* levels, fostering angiogenesis in HCC.

### 2.5 ASH2L‐K312‐Lac Facilitated Angiogenesis in HCC by Modulating H3K4 Methylation in the *VEGFA*‐Encoding Region of the Genome

**Figure 5:**
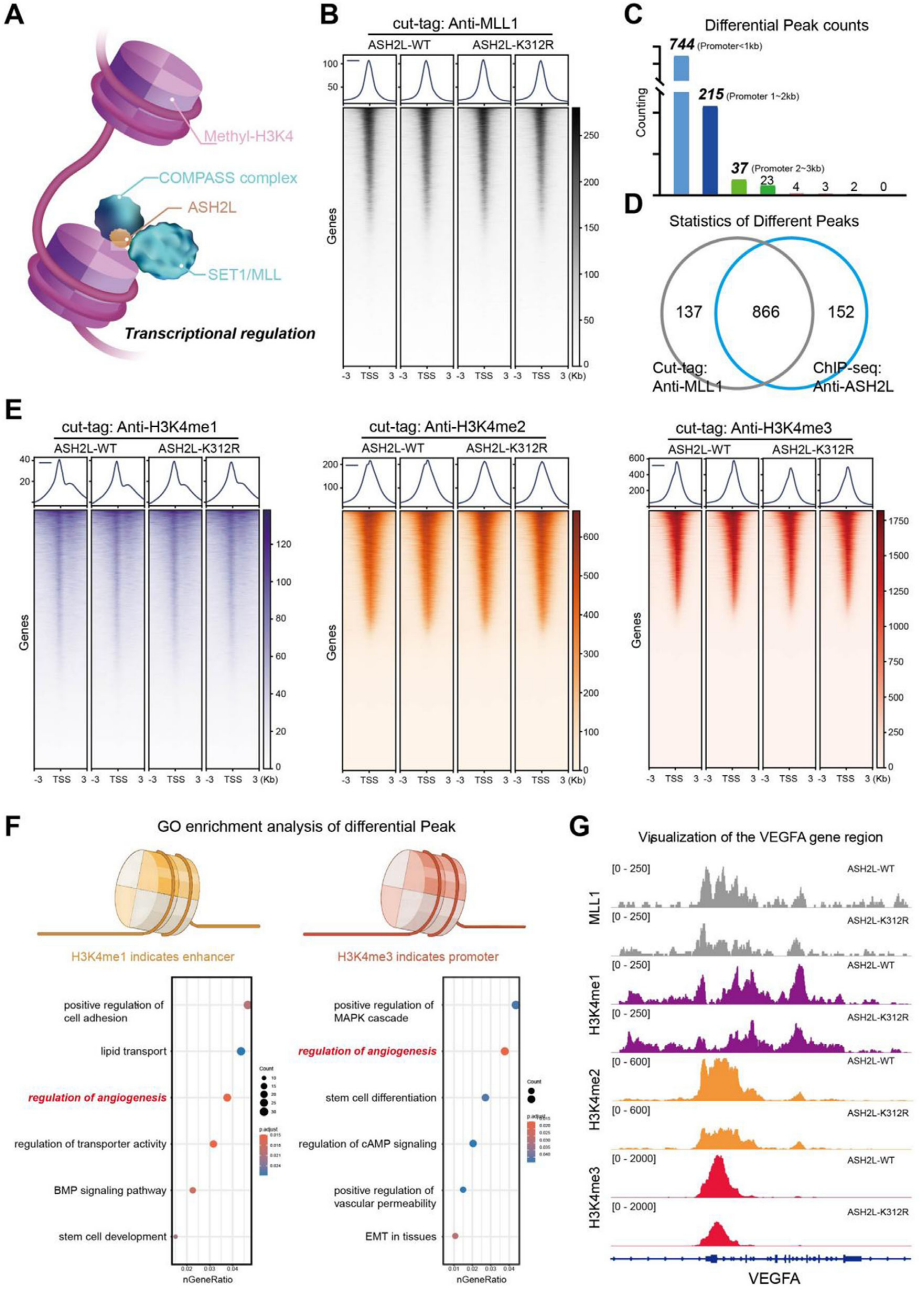
ASH2L‐K312‐lac facilitates angiogenesis in HCC by modulating H3K4 methylation in the *VEGFA*‐encoding region. a) Schematic illustrating the role of ASH2L and its associated COMPASS complex in regulating H3K4 methylation by the MLL complex. b) CUT&Tag (anti‐MLL1) was performed in Huh7‐ASH2L‐WT and Huh7‐ASH2L‐K312R cells. c) Statistical analysis of disparities in MLL1 distribution across the genome before and after ASH2L lactylation loss. d) A high level of concordance was observed between the differentially bound sites on genomic DNA due to ASH2L lactylation and the disparate distribution of MLL1 on the chromatin. e) CUT&Tag (anti‐H3K4me1/2/3) analyses were performed using Huh7‐ASH2L‐WT and Huh7‐ASH2L‐K312R cells. f) Separate analysis of the distribution of H3K4me1/3 in Huh7‐ASH2L‐WT and Huh7‐ASH2L‐K312R cells and clustering analysis showed that angiogenesis was among the top regulated biological processes. g) IGV visualization revealed that ASH2L‐K312‐lac regulated the distribution of MLL1 on the *VEGFA*‐encoding region and modulated H3K4 methylation distribution in that chromatin segment, enhancing *VEGFA* expression in collaboration with promoters and enhancers.

The COMPASS complex functions as a regulatory hub for H3K4 methyltransferases (MLL complex),^[^ ^24^ ^]^ with ASH2L serving as the core subunit essential for coordinating the regulation of H3K4 methylation, a key epigenetic marker closely associated with transcriptional activation ^[^ ^25^ ^]^ (**Figure** **5**A). Because MLL1, an important member of this family, plays a significant role in H3K4 methylation, we selected it for further investigation.^[^ ^26^ ^]^ We used the cleavage under targets and tagmentation (CUT&Tag) method to separately assess MLL1 distribution in the genomes of Huh‐7‐ASH2L‐K312R and Huh‐7‐ASH2L‐WT cells (Figure 5B). The disparate bound sites of MLL1 in the two cell cohorts were situated within the proximal promoter region (Figure 5C). Integration of the differential peaks obtained from the CUT&Tag (anti‐MLL1) and ChIP‐seq (anti‐FLAG‐ASH2L) analyses revealed a high level of concordance between the differentially bound sites on the genomic DNA due to ASH2L lactylation and the disparate distribution of MLL1 on the chromatin (Figure 5D). These findings indicated that ASH2L‐K312‐lac modulated MLL complex distribution across the genome to execute its corresponding biological functions. We also used CUT&Tag technology to assess the spatial distribution of these markers in the two cell groups (Figure 5E). We separately analyzed the distribution of H3K4me1/3 in Huh7‐ASH2L‐WT and Huh7‐ASH2L‐K312R cells and performed clustering analysis on the enhancer and promoter regions specifically targeted for differential expression,^[^ ^27^ ^]^ revealing that angiogenesis was among the top biological processes regulated by their respective functions (Figure 5F). IGV visualization demonstrated that ASH2L‐K312‐lac regulated MLL1 distribution on the *VEGFA‐*encoding segment of the genome and modulated H3K4 methylation distribution in that chromatin segment to enhance *VEGFA* expression in collaboration with promoters and enhancers, thereby promoting angiogenesis in HCC (Figure 5G).

### 2.6 ASH2L‐K312‐Lac is a Promising Target for HCC Clinical Diagnosis and Treatment

**Figure 6:**
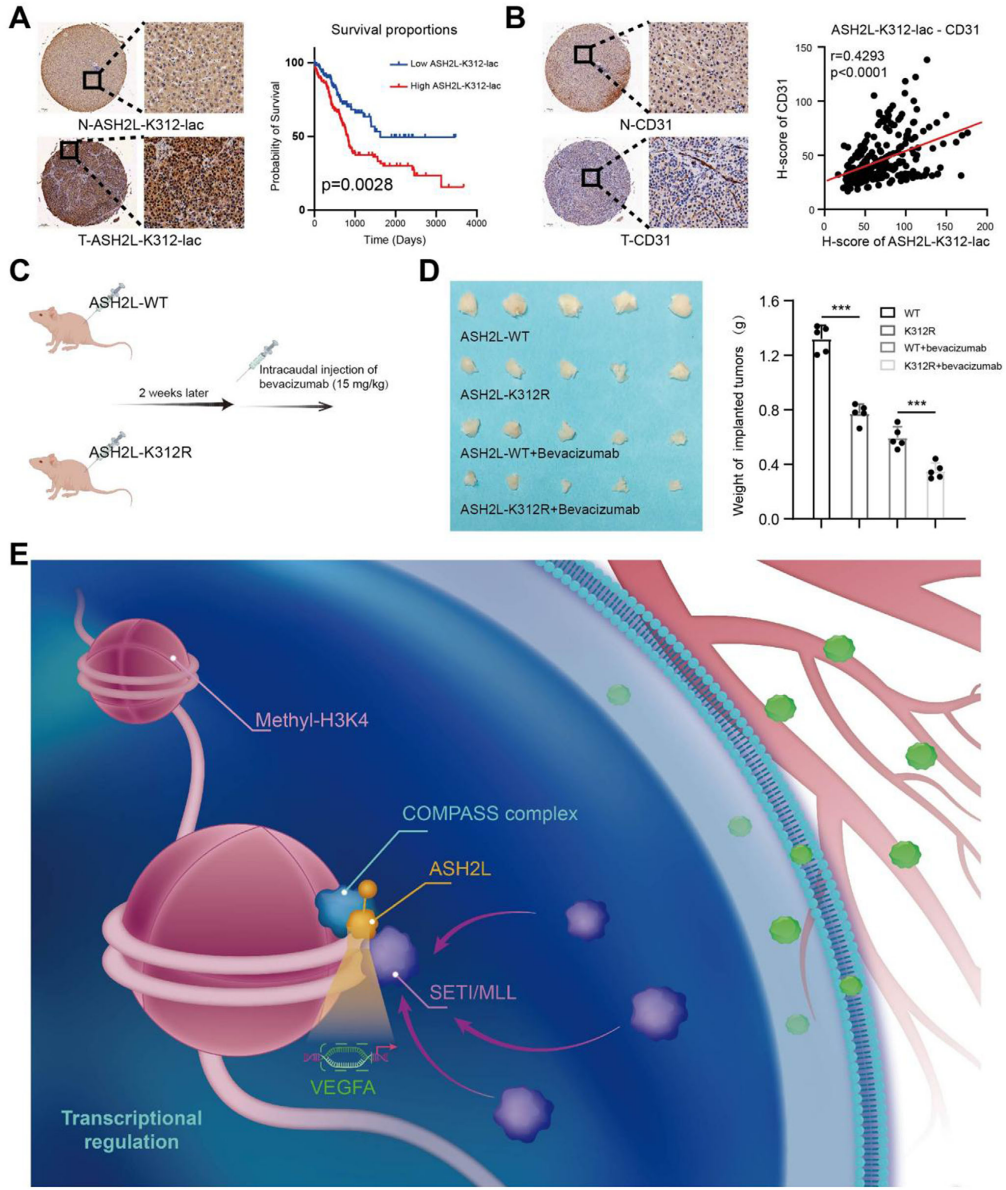
ASH2L‐K312‐lac is a potential target for the future clinical diagnosis and treatment of HCC. a) Previously prepared tissue microarrays (TMAs) used in conjunction with immunohistochemical (IHC) staining revealed increased ASH2L‐K312‐lac levels in HCC, which were negatively correlated with patient prognosis (P = 0.0028). Scale bar: 500/100 µm. b) Using an identical set of TMAs as described above, IHC staining revealed elevated MVD (indicated by CD31 expression) in HCC tumors, along with a positive correlation with ASH2L‐K312‐lac levels (R = 0.4293, P < 0.0001). Scale bar: 500/100 µm. c) Preliminary investigation procedure using a nude mouse subcutaneous tumor model in conjunction with bevacizumab as a pretreatment strategy. d) The pretreatment experiment demonstrated a significantly slower growth rate of tumors with low ASH2L‐K312‐lac levels, accompanied by a notable enhancement in bevacizumab responsiveness. e) Schematic of the model used in this study. ASH2L undergoes lactylation at Lys312. The high expression levels of ASH2L‐K312‐lac in HCC, in conjunction with the associated COMPASS complex, modulate MLL1 distribution on chromatin, facilitating the methylation of the H3K4 residue in the nearby *VEGFA*‐encoding region and leading to enhanced VEGFA expression and secretion in HCC. This process promotes angiogenesis within HCC and accelerates disease progression.

We used tissue microarrays (TMAs) in conjunction with IHC staining to elucidate the role of ASH2L‐K312‐lac in the clinical diagnosis of HCC. ASH2L‐K312‐lac expression levels were higher in tumor tissues than in adjacent tissues (**Figure** **6**A). Furthermore, there was a significant correlation between the expression levels of ASH2L‐K312‐lac and multiple clinical and pathological indicators in patients with HCC (Table S1, Supporting Information), and the level of ASH2L‐K312‐lac expression in the HCC tumor tissues was correlated with patient prognosis (Figure 6A). Similarly, we analyzed the microvascular density (MVD) using an identical set of TMAs, revealing a positive correlation between ASH2L‐K312‐lac expression levels and MVD within tumors, suggesting that ASH2L‐K312‐lac promoted angiogenesis within HCC (Figure 6B).

To further investigate the role of ASH2L‐K312‐lac in the clinical management of HCC, we performed preliminary investigations using nude mouse subcutaneous tumor models in conjunction with bevacizumab (15 mg kg^−1^) pretreatment as the corresponding therapeutic regimen (Figure 6C). Tumors with low ASH2L‐K312‐lac levels showed a slower growth rate, accompanied by enhanced responsiveness to bevacizumab (Figure 6D). These data suggested that ASH2L‐K312‐lac might be a reliable biomarker for assessing the efficacy of antiangiogenic therapy in clinical cases of HCC.

Our findings demonstrated that ASH2L undergoes lactylation at Lys312. Furthermore, high levels of ASH2L‐K312‐lac expression in HCC in conjunction with the COMPASS complex modulated the distribution of MLL1 on the chromatin, facilitating the methylation of the H3K4 residue in the nearby *VEGFA*‐encoding region. Consequently, VEGFA expression and secretion within HCC cells were enhanced, promoting angiogenesis in the tumor tissues and accelerating disease progression (Figure 6E).

## 3 Discussion

HCC is the most prevalent malignant liver tumor worldwide. Its pathogenesis encompasses a complex assortment of molecular events, including gene mutations, epigenetic alterations, and post‐translational protein modifications.^[^ ^28^ ^]^ Among these, protein lactylation is an emerging method of protein modification that, although relatively less studied, has demonstrated considerable potential in HCC research.^[^ ^29^ ^]^ In the clinical management of HCC, the lungs are the most common sites of distant metastasis. Clinicopathological studies have indicated that HCC, a malignancy characterized by abundant microvessels, predominantly promotes intrahepatic and distant organ metastasis via portal systemic circulation. Moreover, lactylation modification in cancer is an independent risk factor for distant metastasis, enhancing the invasion, metastasis, angiogenesis, and immune evasion potential of cancer cells.^[^ ^12^ ^]^

E1A binding protein P300, AARS1, histone lysine acetyltransferase, lysine acetyltransferase 5, and lysine acetyltransferase 7 regulate lactylation within cells. Protein lactylation is catalyzed by lactate dehydrogenase, which facilitates the reaction between lactate and NAD^+^ to produce pyruvate and NADH.^[^ ^30^ ^]^ Within the context of metabolic reprogramming, lactylation modification influences protein function and the cellular metabolic state.^[^ ^31^ ^]^ Elevated lactate levels are often observed in HCC and are potentially associated with increased glycolysis (the Warburg effect) in tumor cells. Protein lactylation is pivotal to HCC development and progression.^[^ ^32^ ^]^ For example, lactylation influences tumor cell migration and invasion by affecting cytoskeletal protein stability or function.^[^ ^33^ ^]^ Furthermore, lactylation alters the tumor microenvironment by affecting pH levels and signaling pathways, promoting tumor growth and angiogenesis.^[^ ^34^ ^]^ However, further comprehensive studies are necessary to elucidate the specific mechanisms, targets, and clinical applications of protein lactylation in HCC.^[^ ^35^ ^]^ Future studies should focus on identifying key target proteins for lactylation, exploring their pathways in HCC development, and developing diagnostic and therapeutic strategies targeting this modification.

In this study, we used lactate‐based mass spectrometry to analyze differentially modified proteins in HCC and adjacent tissues. ASH2L‐K312‐lac was identified as a putative target protein for further investigation. AARS1 was found to be responsible for ASH2L modification and HDAC1 for demodification. ASH2L is integral to the COMPASS complex, regulating chromatin accessibility and gene transcription. It remains unclear whether lactylation modification of ASH2L correlates with the HCC tumor phenotype, and further investigation is warranted. Experiments using conditional Ash2l‐knockout mice indicated the inhibition of HCC progression by Ash2l‐K307R. Further scRNA‐seq analysis of HCC tissues from various mouse cohorts revealed notable discrepancies, particularly among immune and endothelial cells, with pronounced variance evident in the endothelial cell cluster distribution among the experimental subsets. These findings indicate an association between ASH2L lactylation and tumor angiogenesis. To further elucidate the mechanism by which ASH2L lactylation modulates angiogenesis in HCC tumors, we performed transcriptome sequencing of WT and mutant cell lines and analyzed DEGs. VEGFA emerged as a key molecule in tumor angiogenesis. Subsequent dot blot hybridization and rescue experiments validated this conclusion.

The MLL complex comprises six members: MLL1, MLL2, MLL3, MLL4, SET1A, and SET1B. MLL1 plays a key role in H3K4 methylation.^[^ ^36^ ^]^ Thus, it was further examined in this study. ASH2L is an essential constituent of the COMPASS complex and is crucial for regulating chromatin accessibility and gene transcription.^[^ ^37^ ^]^ ChIP‐seq and ATAC‐seq analyses across various cell lines revealed that ASH2L lactylation augmented its binding to DNA regions located within 1 kb upstream of the promoter, facilitating the transcription of genes involved in the *VEGFR*‐mediated angiogenic pathway. Furthermore, a comprehensive analysis integrating ChIP‐seq, ATAC‐seq, and RNA‐seq data identified 11 overlapping genes, with *VEGFR* showing the highest statistical significance. VEGFA is a critical regulatory factor that promotes tumor angiogenesis in HCC. It acts directly on vascular endothelial cells, stimulating their proliferation, migration, and lumen formation, while also increasing vascular permeability. These effects collectively enhance the supply of essential nutrients and oxygen to liver cancer cells, leading to extensive neovascularization and creating a favorable microenvironment for tumor growth and metastasis. In the majority of HCC tissues, VEGFA mRNA expression is markedly upregulated, and this elevated expression positively correlates with increased microvessel density, reflecting active angiogenesis. This angiogenic activity supports the rapid expansion and dissemination of tumor cells. Accumulating evidence demonstrates that VEGFA overexpression is closely associated with enhanced tumor aggressiveness, a greater likelihood of metastasis, and unfavorable clinical outcomes, making VEGFA a valuable biomarker for predicting patient prognosis. Elevated VEGFA levels typically correspond to accelerated tumor progression, an increased risk of recurrence, and reduced overall survival. Based on the findings of this study, we explored the association between lactylation modification and angiogenesis in liver cancer. The results indicated that lactylation modification of ASH2L‐K312, especially in HCC, could promote the malignant progression by up‐regulating the expression and secretion of VEGFA.

In mammals, members of the Set1 family (including MLL1–4) are essential components of the COMPASS complex, which exhibits histone methyltransferase activity.^[^ ^38^ ^]^ MLL proteins, particularly MLL1 (also known as KMT2A), exhibit two primary functions in the COMPASS complex: histone modification and transcriptional regulation.^[^ ^39^ ^]^ Regarding histone modification, MLL proteins, which encompass a SET domain, act as catalysts for H3K4 trimethylation. This modification is prevalent in gene promoter regions, facilitating chromatin structure accessibility for transcription factor binding and subsequently promoting gene transcription activation.^[^ ^40^ ^]^ Regarding transcriptional regulation, MLL regulates the expression of diverse genes by forming distinct complexes via interaction with various proteins, such as transcription factors, chromatin remodeling factors, and RNA‐binding proteins.^[^ ^41^ ^]^ Together, these factors influence the expression patterns of specific genes.^[^ ^42^ ^]^

MLL is also involved in various biological processes, including embryonic development, cell differentiation, and immune responses.^[^ ^43^ ^]^ However, in leukemia and other types of cancer, aberrations, such as gene rearrangements, often lead to dysregulated transcriptional control, promoting tumorigenesis ^[^ ^44^ ^]^ To determine whether MLL function was influenced by ASH2L lactylation, we performed CUT&Tag analysis of MLL in various cell lines. ASH2L lactylation enhanced MLL binding within 1 kb downstream of the DNA promoter region. Furthermore, 80% of the peak binding sites for MLL overlapped with those for ASH2L downstream binding, suggesting that ASH2L lactylation affected MLL binding to downstream DNA, subsequently influencing gene transcription.

MLL1 is a crucial protein catalyst that mediates H3K4 methylation (H3K4me1/2/3) in cells.^[^ ^45^ ^]^ H3K4me1 demarcates active and poised enhancers, H3K4me2 is distributed across the regions of actively expressed genes, and H3K4me3 delineates promoters proximal to transcription start sites and is associated with active gene transcription.^[^ ^46^ ^]^ H3K4me1/3 is the most common archetypal epigenetic modification.^[^ ^47^ ^]^ Examination of H3K4 methylation patterns (H3K4me1/2/3) across enhancers, introns, and promoters confirmed that ASH2L lactylation activated transcription initiation of genes associated with the VEGFR‐mediated angiogenesis pathway via the distribution of H3K4me3. Furthermore, this modification enhanced gene transcription associated with this pathway via the distribution of H3K4me1.

The samples were categorized into high‐ or low‐expression groups based on the protein expression levels of ASH2L‐K312‐lac in HCC tissue chips. Statistical analysis of patients’ clinical data and follow‐up information revealed that those with high levels of ASH2L‐K312‐lac expression had shorter survival times and poorer prognoses. Additionally, antiangiogenic drugs, such as bevacizumab, have been used in HCC treatment with favorable therapeutic outcomes. In this study, we investigated the effects of bevacizumab in combination with a lactate‐modified ASH2L mutation in HCC treatment using a mouse model. The findings demonstrated that this combination exerted the most potent antitumor effect.

In conclusion, our study demonstrated that the lactylated protein ASH2L‐K312‐lac expedited tumor progression by promoting angiogenesis. We determined that its mechanism of action involved modulating the epigenetic factor H3K4me1/3 on DNA via MLL, the key molecule of the COMPASS complex, thereby enhancing the transcriptional activation of genes associated with the angiogenesis signaling pathway mediated by VEGFR. Our findings provide a foundation for targeted drug design against lactylated ASH2L. However, further comprehensive studies are necessary to substantiate our hypothesis.

## 4 Experimental Section

### Cell Culture

Human HCC cell lines (Huh‐7 (RRID:CVCL_0336) and Hep3B (RRID:CVCL_0326)) and HUVECs (RRID:CVCL_E5ZU) were procured from Shanghai Anwei Biotechnology Co., Ltd at 2022‐06. The HCC cell lines were cultured in Dulbecco modified Eagle medium supplemented with 10% fetal bovine serum. The HUVECs were maintained in a specialized endothelial cell culture medium (ScienCell). All cells underwent short tandem repeat identification and were regularly tested for the presence of *Mycoplasma* spp. Simultaneously, the rigorously controlled cultivation environment ensures that the cells remain free from contamination by any potential pathogens.

### Antibodies and Reagents

The following antibodies were used in our experiments: anti‐L‐lactyl lysine rabbit mAb (WM103, Micrometer Biotech Company, RRID:AB_3697039), anti‐MLL1 (14689, Cell Signaling Technology, RRID:AB_2688009), anti‐ASH2L (39099, Active Motif; 1:2000 for western blotting, RRID:AB_2793605), anti‐H3K4me1 (A22078, Abclonal, RRID:AB_2764315), anti‐H3K4me2 (A22143, Abclonal, RRID:AB_2764316), anti‐H3K4me3 (9751, Cell Signaling Technology, RRID:AB_2616028), anti‐FLAG (F1804, Sigma; 5 µg reaction^−1^ for ChIP‐seq and co‐IP; 1:5000 for western blotting, RRID:AB_262044), anti‐Myc‐tag (16286‐1‐AP, Proteintech; 5 µg reaction^−1^ for co‐IP; 1:2000 for western blotting, RRID:AB_11182162), anti‐β‐actin (66009‐1‐Ig, Proteintech; 1:5000 for western blotting, RRID:AB_2687938), anti‐AARS1 (17394‐1‐AP, Proteintech; 1:1000 for western blotting; 5 µg reaction^−1^ for co‐IP, RRID:AB_2219748), anti‐EP300 (54062, Cell Signaling Technology; 1:1000 for western blotting, RRID:AB_2799450), anti‐KAT5 (A1678, Abclonal; 1:1000 for western blotting, RRID:AB_2763733), anti‐KAT7 (A5823, Abclonal; 1:1000 for western blotting, RRID:AB_2766575), anti‐KAT8 (A3390, Abclonal; 1:1000 for western blotting, RRID:AB_2863049), and anti‐HDAC1 (A19571, Abclonal; 1:1000 for western blotting, 5 µg/reaction for co‐IP, RRID:AB_2862675). Lactic acid (HY‐B2227) was obtained from MedChemExpress. To establish a range of concentration and time gradients, lactic acid was added to the culture medium at final concentrations of 0, 20, 50 and 100 mm, and cultured the cells for 12 hours. Furthermore, to evaluate the effect of treatment duration, lactic acid was added at a final concentration of 100 mm and cultured the cells for 6, 12 and 24 hours, respectively.

The preparation procedure of the ASH2L‐K312‐lac‐specific antibody was briefly outlined below. For detailed protocols and analytical methods, please refer to the supplementary material “ASH2L‐K312‐lac‐specific Antibody Preparation Protocol”. ASH2L‐K312‐lac‐specfic antibody was generated by Hua'an Biotechnology (Hangzhou, China). The ASH2L‐K312‐lac peptide (CGGTTGTTK(lac)KARSDP) was first coupled to keyhole limpet hemocyanin and BSA, followed by multiple subcutaneous injections to immunize New Zealand white rabbits. After immunization, blood was collected from the rabbits and the presence of ASH2L‐K312‐lac‐specific antibodies in the serum was evaluated using enzyme‐linked immunosorbent assay (ELISA). For final antibody purification, the serum was eluted through the ASH2L‐K312‐lac peptide column and then passed through a non‐ASH2L‐K312‐lac peptide column to obtain the specific antibody.

### Collection of Human HCC Samples

This study was approved by the Ethics Review Board of the Affiliated Taizhou People's Hospital of Nanjing Medical University (2022‐008‐01). HCC specimens were collected and matched adjacent noncancerous tissues from patients at the Affiliated Taizhou People's Hospital of Nanjing Medical University (China) diagnosed with HCC who had not received any preoperative cancer treatment. All study participants gave informed consent.

### Western Blotting

Cell extracts and fresh tissues were prepared on ice in RIPA cell lysis buffer (Beyotime, Shanghai, China) with 1% protease inhibitor (Beyotime) for 20 min. The proteins were quantified using BCA kits (Biosharp, Wuhan, China). Then equal amounts were separated by sodium dodecyl sulfate‐polyacrylamide gel electrophoresis and transferred onto polyvinylidene fluoride membranes. The membranes were blocked with 5% milk in Tris‐buffered saline with Tween 20 for 1 h at room temperature and incubated with a primary antibody at 4 °C overnight. After washing, the corresponding secondary antibodies were added. The immunoblots were visualized using ECL reagents (Biosharp), and images were recorded using a chemiluminescent image system (Bio‐Rad).

### RNA Extraction and Reverse Transcription qPCR

Total RNA was extracted from the tissue samples using RNA‐easy Isolation Reagent (R701, Vazyme, Nanjing, China) according to the manufacturer's guidelines. mRNA was quantified using a spectrophotometer (NanoDrop) and reverse‐transcribed it into cDNA. Furthermore, qPCR assays were performed in triplicate using AceQ Universal SYBR qPCR Master Mix (Q511, Vazyme) with primer pairs targeting VEGF

(RT‐VEGF‐F: CATCACCATGCAGATTATGCG and RT‐VEGF‐R: CTATCTTTCTTTGGTCTGCATTCAC) and β‐actin (RT‐β‐actin‐F: TCCCTGGAGAAGAGCTACG and RT‐β‐actin‐R: GTAGTTTCGTGGATGCCACA). The relative mRNA abundance was calculated using the comparative C_T_ method and normalized to β‐actin.

### IHC Staining and Multiplexed Immunofluorescence Assays

To prepare for IHC staining, TMA sections and mouse liver tissue samples were routinely dewaxed and rehydrated. Endogenous peroxidase activity was blocked in the dark for 10 min, followed by citrate‐based antigen retrieval at 95 °C and blocking of nonspecific binding with 5% BSA for 1 h at room temperature. The TMA sections were incubated overnight with the primary antibodies in a humidified chamber at 4 °C. After incubation with biotin‐labeled secondary antibodies, staining was visualized using a DAB Substrate Kit (Vector Laboratories). The mouse liver tissue samples were stained by Wuhan Sercivebio Biotechnology Co., Ltd. using the Opal Multiplex Fluorescence Staining System (PerkinElmer).

### Animal Models and Adenovirus

C57BL/6 mice (male, 2 weeks old) and BALB/c athymic nude mice (male, 4–6 weeks old) were obtained from the Laboratory Animal Center at Nantong University. WT and gene‐knockout mice on a C57BL/6 background were obtained from the Guangzhou Cyagen Company. All animal experiments were performed according to the National Institutes of Health guidelines and approved by the Ethics Committee of the Laboratory Animal Center of Nantong University (RDD number: S20230420‐007). The mice were maintained and fed in a pathogen‐free vivarium under standard conditions following the guidelines for animal care.

We created a mouse model of HCC by administering 25 mg/kg DEN (Sigma‐Aldrich) via intraperitoneal injection to 14‐day‐old male C57BL/6 mice to induce HCC, followed by intraperitoneal injections of CCl_4_ (0.5 mL/kg dissolved in corn oil) once a week for 20 weeks. After 2 weeks, the mice were randomly assigned to the control or experimental group. Upon experiment completion, the DEN/CCl4‐treated mice were euthanized and dissected to collect the liver tumor tissues for subsequent analysis.

The adenovirus used in this study was obtained from Jiangsu Jianweipu Technology Co., Ltd. Because the virus was intended for injection into mice, a mutant virus was chosen featuring the K307 site derived from the human homologous Ash2l gene in mice.

### CUT&Tag Assay

The following antibodies were used for the CUT&Tag assay: anti‐MLL1 (1 µL/reaction), anti‐H3K4me1 (1 µg/reaction), anti‐H3K4me2 (1 µg/reaction), and anti‐H3K4me3 (1 µL/reaction). The CUT&Tag assay for anti‐MLL1 was performed using the NovoNGS® CUT&Tag 4.0 High‐Sensitivity Kit (for Illumina®) (N259‐YH01, Novoprotein, Shanghai, China). For H3K4me1/2/3, the CUT&Tag Assay Kit (pAG‐Tn5) was used for Illumina (RK20265, Abclonal). All relevant experiments were performed according to the manufacturer's instructions and quality control requirements. The resultant DNA libraries were sequenced by Nanjing Jiangbei New Area Biopharmaceutical Public Service Platform Co., Ltd.

### High‐Throughput Sequencing

scRNA‐seq was performed using an ABcellar Single Cell 3' RNA‐seq Lib Prep Kit (RK20390, Abclonal). ChIP‐seq was performed using a Sonication ChIP Kit (RK20258, Abclonal), and the library was constructed using a Scale ssDNA‐seq Lib Prep Kit for Illumina V2 (RK20228, Abclonal).

ATAC‐seq was performed using a Hyperactive ATAC‐Seq Library Prep Kit for Illumina (TD711, Vazyme). Bulk mRNA‐seq was polished using a VAHTS Universal V10 RNA‐seq Library Prep Kit for Illumina (NR606, Vazyme).

All relevant experiments were performed according to the manufacturer's instructions and quality control requirements. The complete DNA library was sequenced by Nanjing Jiangbei New Area Biopharmaceutical Public Service Platform Co., Ltd.

All sequencing in this study was performed using a NovaSeq 6000 platform. For the ChIP‐seq, Fc‐CUT&Tag, CUT&Tag, and ATAC‐seq experiments, the raw sequencing data were first subjected to quality control using FastQC (v0.12.1), followed by adapter trimming with Trim Galore (v0.6.10). The cleaned reads were aligned to the reference genome using Bowtie2 (v2.5.4). Duplicate reads were removed using Sambamba (v1.0.1). Peak calling was performed using MACS2 (v2.1.4) and differential peak analysis using DiffBind (v3.14.0).

For mRNA‐seq data, initial quality control and adapter trimming were performed using FastQC and Trim Galore, respectively, followed by alignment using HISAT2 (v2.2.1). Gene counts were generated using Subread (v2.0.6), and differential expression analysis was performed using DESeq2 (v1.44.0).

The scRNA‐seq data were aligned using Cell Ranger (v8.0.1). Downstream analysis, including sample integration and clustering, was performed using Seurat (v5.0.0), with additional functional analysis using tidyselect (v1.2.1) and CellChat (v1.6.1). All analyses were performed using the default parameters of each software tool unless otherwise specified.

The DEGs in the bulk RNA‐seq data were identified using the DESeq2 package in R, the DEGs were selected according to a combined criterion of adjusted P < 0.05 and |log2FC| >1 in both bulk RNA‐seq analyses, while the DEGs from the scRNA‐seq data were identified using the FindMarkers function in the Seurat package, the DEGs were selected according to a combined criterion of logfc.threshold = 1,# min.pct = 0.2 in scRNA‐seq andanalyses. Specifically, 545 upregulated and 223 downregulated genes were identified in the malignant hepatocytes of the ASH2L‐K312‐lac group compared with the control group. The downregulated genes in the malignant hepatocytes of the ASH2L‐K312‐lac group underwent pathway enrichment analysis. Enriched pathways were identified by the hyper‐geometric test with false discovery rate (FDR) correction (FDR < 0.05).

### Integrative Analysis of Bulk RNA‐seq, ATAC‐seq, and ChIP‐seq Data

Peaks derived from the ChIP‐seq and ATAC‐seq data were imported into R using the DiffBind package (a combined criterion of FDR < 0.05 & Fold >1). For bulk RNA‐seq data, DEGs were determined using the DESeq2 package with an adjusted P‐value < 0.05 and a minimum two‐fold change. Differentially accessible peaks were annotated to gene symbols using the ChIPseeker package in R. 1,032 and 828 genes were identified with differential chromatin accessibility from the ChIP‐seq and ATAC‐seq data, respectively. In comparison, bulk RNA‐seq analysis identified 873 DEGs. Integrative multiomics analysis revealed 11 genes exhibiting both differential expression and chromatin accessibility.

### Tube Formation Assay, Matrigel Plug Assay, and Proteome Profiler Human Angiogenesis Antibody Array Profiling

The tube formation and Matrigel plug assays were performed as previously described.^[^ ^48^ ^]^ For the tube formation assay, a 96‐well plate coated was incubated with Matrigel (354234, Corning Life Sciences) at 37 °C for 30 min. Prior to this, HUVECs were starved overnight and cultured in different groups of supernatants for 24 h. The HUVECs (2 × 10^4^) were suspended in 100 µl of the corresponding supernatant group and placed in a precoated 96‐well plate. After incubation at 37 °C for another 6 h, micrographs were captured.

For the Matrigel plug assay, male BALB/c athymic nude mice (4–6 weeks old) were subcutaneously injected on the dorsal side with 500 µl of Matrigel mixed with the corresponding cell culture supernatant. All animal experiments were approved by the Institutional Animal Care and Use Committee of Nantong University. Two Matrigel plugs were implanted in each mouse. After 5 days, the mice were euthanized.

A Proteome Profiler Human Angiogenesis Antibody Array Kit (ARY007, R&D Systems) was used to detect changes in cytokine levels in the cell supernatants. All experiments were performed according to the manufacturer's instructions and quality control requirements.

### Statistical Analysis

All statistical analyses were performed using GraphPad Prism 9.5 software. Student's *t*‐test was used to determine the significance between groups. Overall survival was estimated using the Kaplan–Meier method, and the log‐rank test was used to assess significance. The Youden index was used to classify patients into low‐ or high‐ASH2L‐K312‐lac expression groups in the survival analysis. Image‐Pro Plus 6 was used to quantify the intensity of ASH2L‐K312‐lac staining of CD31, and their correlations were analyzed using the chi‐square test (Fisher exact test). P‐values < 0.05 were considered statistically significant.

### Ethics Approval Statement and Patient Consent Statement

All animal experiments were approved by the Committee on Ethics of the Laboratory Animal Center of Nantong University (RDD number: S20230420‐007).

The written informed consent was collected from the patients, and the experimental protocol got permission from the institutional ethics review board of The Affiliated Taizhou People's Hospital of Nanjing Medical University (2022‐008‐01).

### Ethics approval statement

This study was approved by the Ethics Review Board of the Affiliated Taizhou People's Hospital of Nanjing Medical University (2022‐008‐01). HCC specimens were collected and matched adjacent noncancerous tissues from patients at the Affiliated Taizhou People's Hospital of Nanjing Medical University (China) diagnosed with HCC who had not received any preoperative cancer treatment.

### Patient Consent Statement

All study participants provided informed consent.

### Funding

This project was supported by the National Natural Science Foundation of China (Grant 82372746, 81873094), China Postdoctoral Science Foundation (Certificate Number: 2024M762336), The Youth Fund of Taizhou People's Hospital Affiliated to Nanjing Medical University (TZKY20240109), Scientific research start‐up fund of Taizhou People's Hospital (QDJJ202103, QDJJ202106), Taizhou Society Development Project, Jiangsu, China (TS202306, TS202308), General Project of Jiangsu Provincial Health Commission (H2023030, H2023132), Key research project of Taizhou School of Clinical Medicine, Nanjing Medical University(TZKY20230306), Research Program of Taizhou School of Clinical Medicine, Nanjing Medical University (TZKY20230209), Medical Research Project of Jiangsu Provincial Health Commission (K2023074), The Wuxi Health Commission Youth Project (grant numbers Q202245), the Jiangsu Innovative Healthcare Team(No.［2023］31)

## Conflict of Interest

The authors declare no conflict of interest.

## Supporting information



Supporting Information

## Data Availability

The data that support the findings of this study are available from the corresponding author upon reasonable request.
