# Supplementary material for: ASH2L‐K312‐Lac Stimulates Angiogenesis in Tumors to Expedite the Malignant Progression of Hepatocellular Carcinoma
Source: Adv Sci (Weinh). 2025 Jul 29;12(40):e09477. doi: 10.1002/advs.202509477 (PMC12561209; doi:10.1002/advs.202509477)
Supplement: Supplementary file 1 — Supporting Information [file ADVS-12-e09477-s001.docx]

**ASH2L-K312-lac stimulates angiogenesis in tumors to expedite the malignant progression of hepatocellular carcinoma**

Hexu Han^1,*,#^, Shuai Wang^2,*^, Lixing Ma^3,*^, Haimeng Yin^4,*^, Xinxiang Cheng^5,*^, YiFan Wang^1^, Suqin Xia^1^, Yi Zhang^1^, Yue Zhang^2^, Rong Zhu^6^, Cuixia Liu^1,#^, Dakun Zhao^1,#^ Xiangqian Gu^6,#^ He Zhu^7,#^, Yin Yuan^8, 9, #^

1. Department of Gastroenterology, The Affiliated Taizhou People's Hospital of Nanjing Medical University, Taizhou School of Clinical Medicine, Nanjing Medical University, Taizhou, Jiangsu 225300, People's Republic of China.
2. Clinical Medical Laboratory Center, The Affiliated Taizhou People's Hospital of Nanjing Medical University, Taizhou, Jiangsu 225300, China.
3. Department of Hepatobiliary Surgery, Changzhi People's Hospital, The Affiliated Hospital of Changzhi Medical College, No. 502 Changxing Middle Road, Changzhi, Shanxi 046000, China.
4. Department of Otorhinolaryngology Head and Neck surgery, Affiliated Hospital of Nantong University, Medical School of Nantong University, Nantong 226001, China.
5. Department of General Surgery of the Wuxi NO.2 Chinese Medcine Hospital, Wuxi, Jiangsu, China.
6. Department of Hepatobiliary Surgery, The Affiliated Wuxi People’s Hospital of Nanjing Medical University, Wuxi People’s Hospital, Wuxi Medical Center, Nanjing Medical University, China
7. Drug Clinical Trial Center, The Affiliated Taizhou People's Hospital of Nanjing Medical University, Taizhou 225300, China
8. Department of Hepatobiliary Surgery, The Affiliated Taizhou People's Hospital of Nanjing Medical University, Taizhou School of Clinical Medicine, Nanjing Medical University, Taizhou, Jiangsu 225300, People's Republic of China.
9. Lead contact


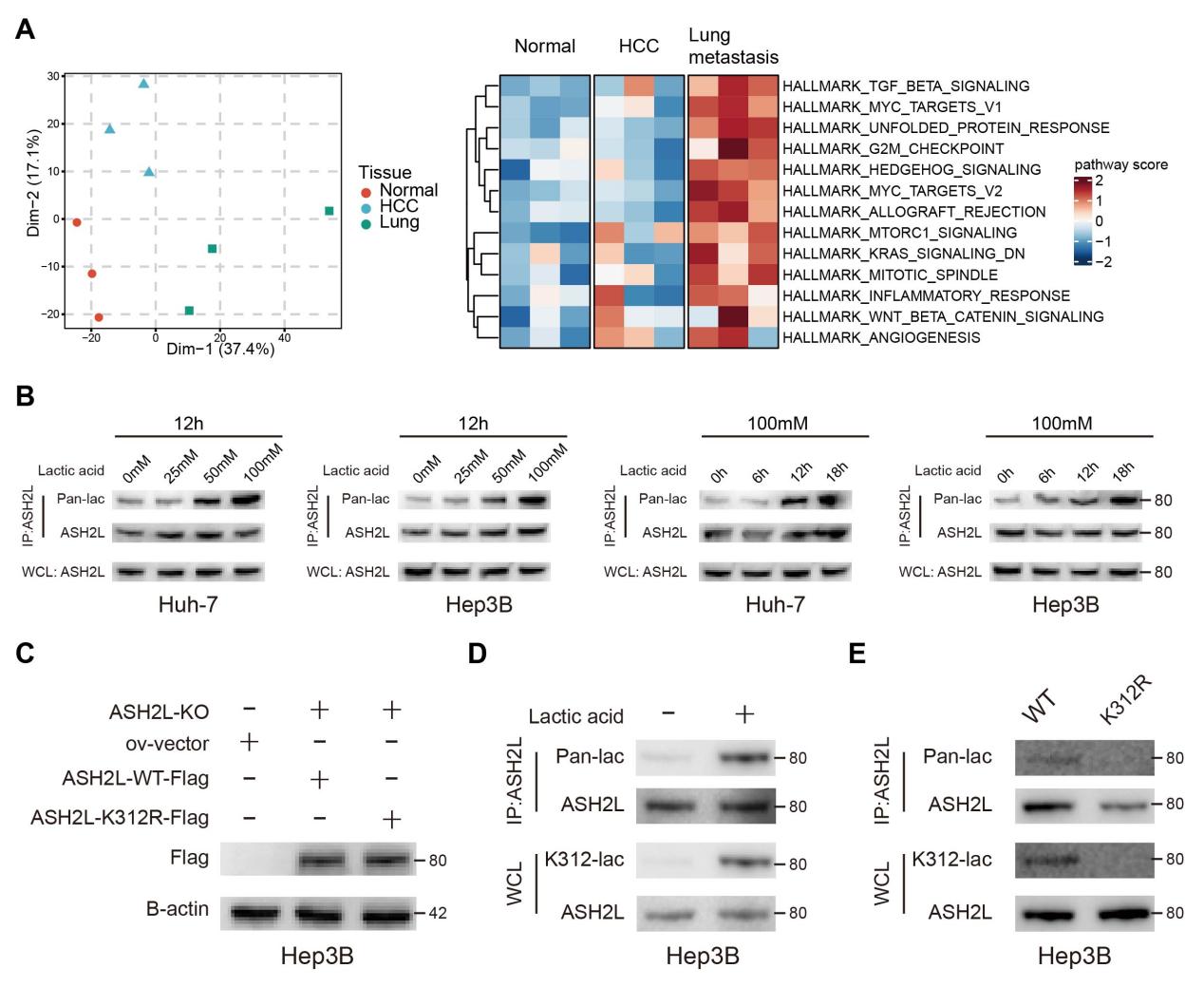


**Supplement Figure 1.** **Lysine-312 is the key residue that mediates the Lactylation in ASH2L.**

1. the lactylation profiles across these three tissue types exhibited distinct patterns (left), hallmark oncogenic pathways were significantly upregulated in the primary and metastatic lesions, suggesting a potential role of protein lactylation in promoting HCC progression and distant metastasis (right).
2. By establishing diverse time and concentration gradients, specifically, we added lactic acid to the culture medium at final concentrations of 0, 20, 50 and 100 mM respectively, and then continued to culture the cells for 12 hours. Additionally, after adding lactic acid at a final concentration of 100 mM to the culture medium, we continued to culture for 6, 12 and 24 hours respectively to evaluate the impact of time on the experimental results, we discovered that ASH2L truly undergoes lactylation modification.
3. Western blot assays are employed for the detection of successful ASH2L-WT and ASH2L-K312R cells construction.
4. After the addition of lactate (100mM, 12h) to the culture medium, Western blotting and IP assays revealed a significant increase in both the total Lactylation level and the corresponding lysine-312 Lactylation level of ASH2L.
5. After conducting Western blotting and IP assays, a significant reduction in the total Lactylation and lysine-312 autosomal Lactylation levels of the ASH2L protein was observed in ASH2L-K312R cells compared to ASH2L-WT cells.


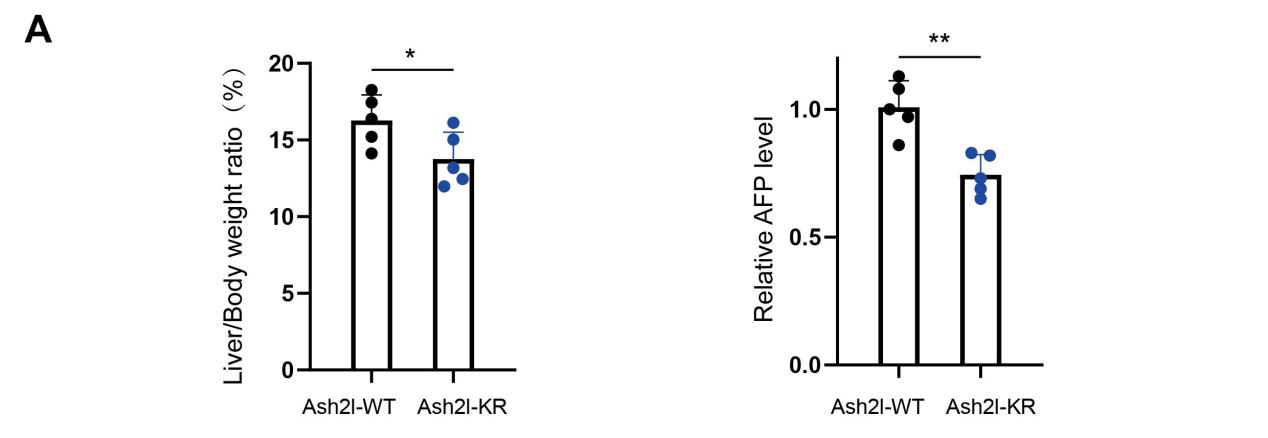


**Supplement Figure 2. Lactylation of ASH2L facilitates the malignant progression of HCC by promoting the generation of vascular endothelial cells**

1. In comparison to the Ash2l-WT mic, the mutant group of mice exhibited a significant reduction in tumor size and number following chemical induction of the tumor model, (n=5/group).


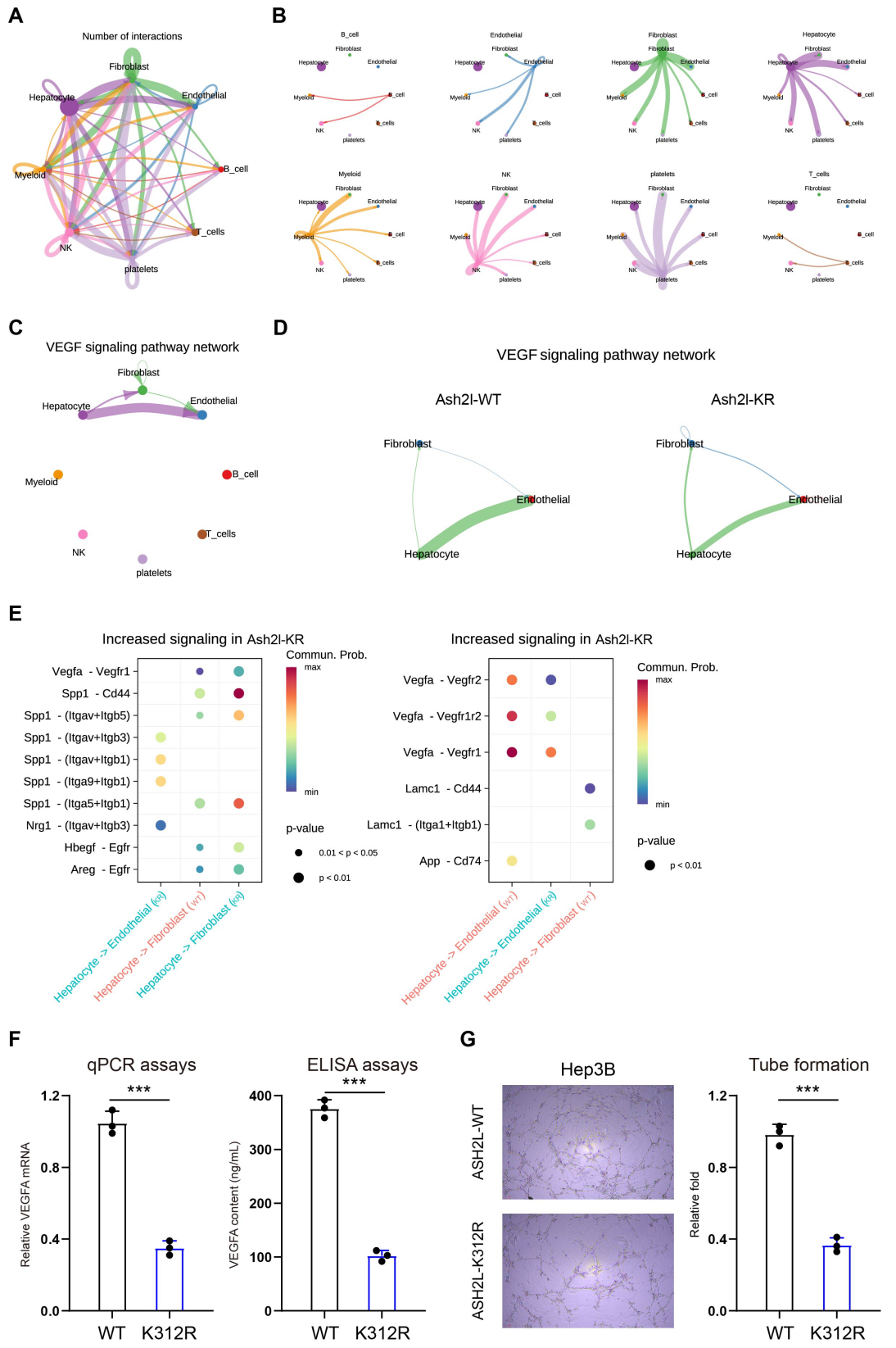


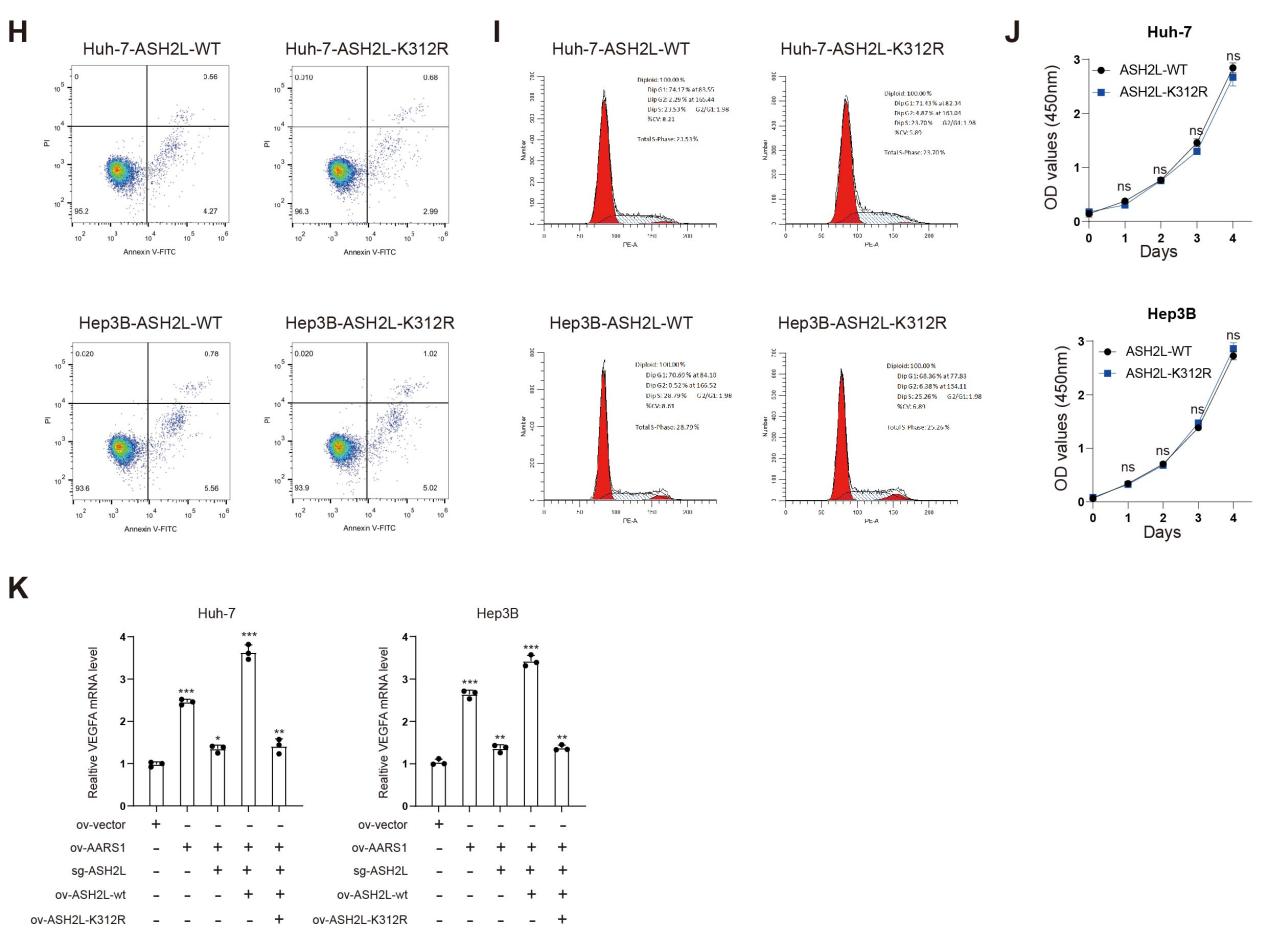


**Supplement Figure 3. Lactylation of ASH2L impacts angiogenesis in HCC tumor cells via VEGFA**

1. The CellChat analysis was employed to systematically investigate the patterns and conditions of intercellular interactions in this study.
2. Systematically illustrate the relationships of cell interactions among different cell types in this study.
3. Systematically demonstrate the interactions of the VEGF signaling pathway among distinct cell subpopulations in this study.
4. Ash2l-KR significantly reduced the VEGFA signaling activity of hepatocytes and its impact on endothelial cells (P < 0.05).
5. The interactions between Vegfa and its receptors (Vegfr1/Vegfr1r2/Vegfr2) were significantly reduced in Ash2l-KR compared to Ash2l-WT
6. qPCR and ELISA results reveal diminished VEGFA expression and secretion with K312R mutation in Hep3B cells.
7. In the Tube formation experiment, the angiogenic potential of HUVECs to form capillary-like structures in the supernatant of Hep3B-ASH2L-K312R cells was significantly lower compared to that of the Hep3B-ASH2L-WT group.

H-K. Utilizing previously established cell lines, we found that AARS1 could regulate the expression levels of VEGFA in HCC cells. However, ASH2L-lys312 had minimal effect on cell cycle, apoptosis, and proliferation of tumor cells. Moreover, mutation at the 312th position of ASH2L significantly diminishes the ability of AARS1 to enhance VEGFA expression in HCC cells, indicating that AARS1 promotes angiogenesis in liver cancer through ASH2L lactylation.


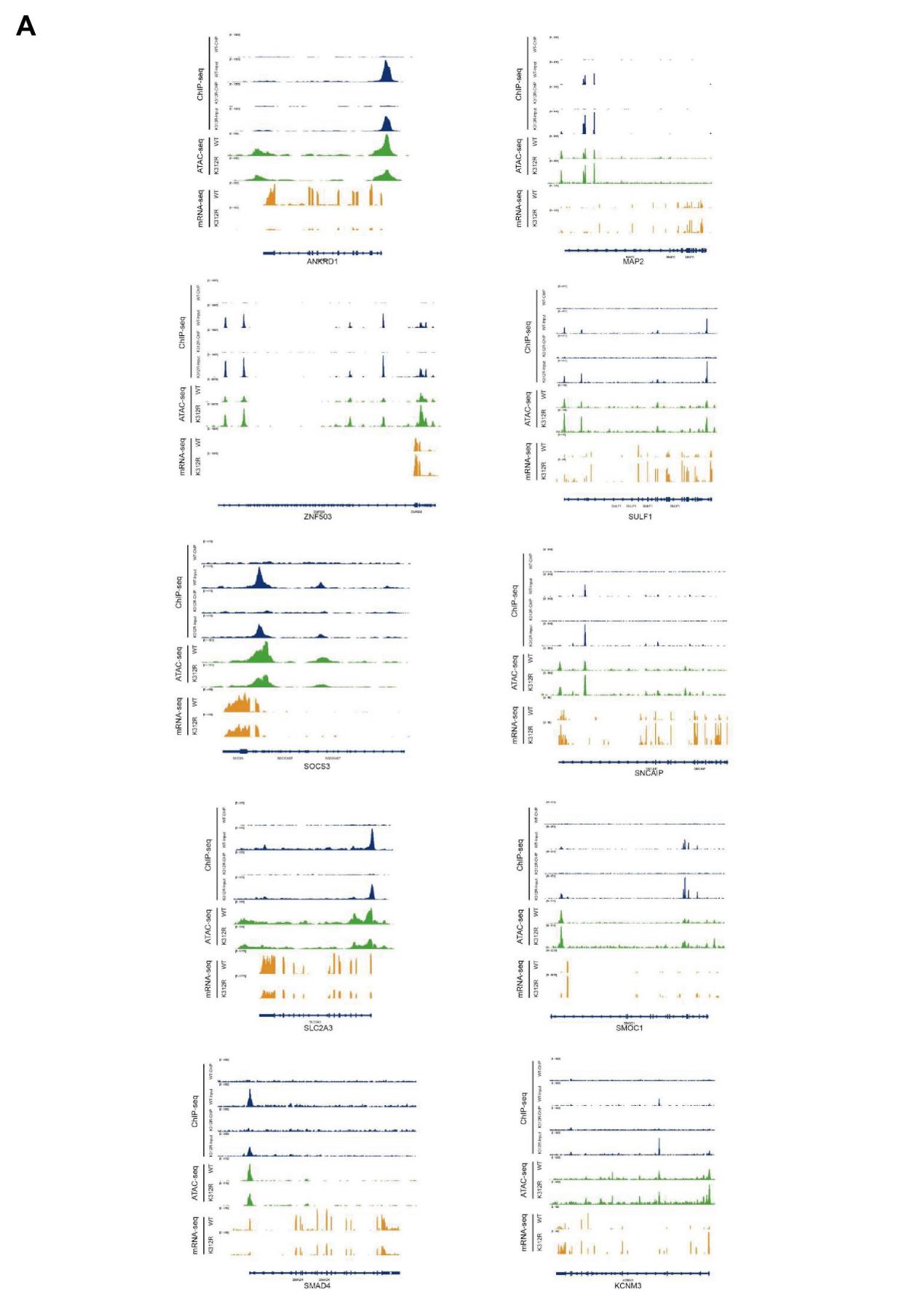


**Figure 4. ASH2L-K312-lac showed an increased affinity for the *VEGFA* gene, facilitating its transcription.**

1. IGV results showed the visualization results of the other ten genes in the combined analysis. Regarding other molecules, such as SOCS3 and SMAD4A, while they are significant genes involved in malignant progression of the tumor, their direct correlation with the formation of new blood vessels within the tumor is not as prominent, which is why they were not selected.

| Variables | Expression of ASH2L-K312-lac | | *P* value |
| --- | --- | --- | --- |
|  | low (120) high (120) | |  |
| Sex  Female  Male | 65  55 | 62  58 | 0.6975 |
| Age，y  ≤51  >51 | 49  71 | 61  59 | 0.1200 |
| Preoperative AFP (ng/mL)  ≤20  >20 | 52  68 | 64  56 | 0.1211 |
| HBsAg  Negative  Positive | 50  70 | 75  45 | **0.0012** |
| Liver cirrhosis  No  Yes | 41  79 | 19  101 | **0.0010** |
| Tumor size(cm)  ≤5  >5 | 73  47 | 52  68 | **0.0067** |
| Tumor number  Single  Multiple | 96  24 | 89  31 | 0.2823 |
| Tumor differentiation  I-II  III-IV | 86  34 | 67  53 | **0.0107** |

**Table S1. Correlation between ASH2L-K312-lac expression and clinical data of patients with HCC (# *P* value < 0.05 is considered as statistically significant).**
